# MIAMI––a tool for non-targeted detection of metabolic flux changes for mode of action identification

**DOI:** 10.1093/bioinformatics/btaa251

**Published:** 2020-04-23

**Authors:** Christian-Alexander Dudek, Carsten Reuse, Regine Fuchs, Janneke Hendriks, Veronique Starck, Karsten Hiller

**Affiliations:** b1 Department of Bioinformatics and Biochemistry, Technische Universität Braunschweig, Braunschweig 38106, Germany; b2 BASF Metabolome Solutions GmbH, Berlin 10589, Germany; b3 BASF SE, Lampertheim 68623, Germany; b4 Department of Immunometabolism, Helmholtz Center for Infection Research, Braunschweig 38124, Germany

## Abstract

**Summary:**

Mass isotopolome analysis for mode of action identification (MIAMI) combines the strengths of targeted and non-targeted approaches to detect metabolic flux changes in gas chromatography/mass spectrometry datasets. Based on stable isotope labeling experiments, MIAMI determines a mass isotopomer distribution-based (MID) similarity network and incorporates the data into metabolic reference networks. By identifying MID variations of all labeled compounds between different conditions, targets of metabolic changes can be detected.

**Availability and implementation:**

We implemented the data processing in C++17 with Qt5 back-end using MetaboliteDetector and NTFD libraries. The data visualization is implemented as web application. Executable binaries and visualization are freely available for Linux operating systems, the source code is licensed under General Public License version 3.

## 1 Introduction

Cellular metabolism can quickly adapt to exogenous stimuli. For this reason, the identification of metabolic flux changes in biological systems is of high value in various scientific fields, like health, nutrition, pharmacology, biotechnology and chemistry. During the last decades, stable-isotope labeling in combination with mass spectrometry, has emerged as a powerful tool to gain insights into biochemical reaction mechanisms and for the determination of metabolic fluxes (Birkemeyer *et al.*, 2005). Mass isotopomer distributions (MIDs) or isotopic enrichment patterns of target metabolites downstream of a tracer can be determined computationally from mass-spectrometric data and are indicative of metabolic fluxes in the system (Buescher *et al.*, 2015). While most approaches focus on targeted metabolic flux analysis (Antoniewicz *et al.*, 2007), some methods and algorithms have been developed for non-targeted or global analysis of metabolic fluxes in stable-isotope labeling experiments (Hiller *et al.*, 2010, 2011). While targeted approaches allow for the specific analysis of metabolites and linked fluxes with high accuracy, the power of non-targeted approaches is the discovery of previously unconsidered metabolites even without a detailed *a priori* knowledge of the biological system (Bueschl *et al.*, 2017; Creek *et al.*, 2012). However, non-targeted acquired data need to be put into a metabolic context to reveal insights on metabolic flux changes, which is still a challenging task (Schrimpe-Rutledge *et al.*, 2016). One approach is to connect metabolites with similar MIDs to create a MID similarity network (Weindl *et al.*, 2016a).

Today, various software tools and packages are available for data analysis of either targeted or non-targeted datasets of stable isotope labeling experiments (Misra, 2018). However, all these tools either focus on targeted or non-targeted methods exclusively, without considering the strength of the other method. While the discovery of previously unconsidered metabolic interactions is quite important, it is also essential to link these data to the current biological knowledge. To address this shortcoming, we developed mass isotopolome analysis for mode of action identification (MIAMI) as a comprehensive tool for the detection, analysis and visualization of global metabolic flux changes. Our software considers all detectable known and unknown metabolites in GC-MS datasets and detects MID changes between different conditions. Based on the algorithms of MIA (Weindl *et al.*, 2016b), NTFD (Hiller *et al.*, 2013) and MetaboliteDetector (Hiller *et al.*, 2009), we extended the contextualization beyond pure non-targeted MID-based analysis. Using pre-defined reference pathways and mass-spectral reference libraries, we can map non-targeted MID data into a broader biological context. Finally, MIAMI reveals metabolic flux changes between different conditions even in *a priori* unknown metabolites and thus identifies potential metabolic targets.

As experimental prerequisites, for each condition under investigation, a set of stable-isotope labeled and unlabeled extracts of cellular metabolites is required. Detailed experimental procedures are described in Weindl *et al.* (2016a).

## 2 Features

MIAMI offers the full computational pipeline from raw GC-MS data in netCDF format to a final network contextualization and data visualization and can export all results in various formats. In addition, data in MetaboliteDetector format can be directly imported for further analysis. MIAMI takes advantage of the NTFD algorithm (Hiller *et al.*, 2010, 2013) to detect labeled metabolites and determines MIDs for all detected metabolites and all conditions. MIDs are then used to create a similarity network and to identify variability between investigated conditions (Weindl *et al.*, 2016a,b). Optionally, metabolites can be identified using reference spectra libraries.

Variability of MIDs reflect altered metabolic fluxes and are the basis to detect changes between the conditions. If mass isotopolog abundances change more than a definable variability threshold, the underlying flux is considered as changed and is differentially colorized within the pathway. A connection between two metabolites is considered as a potential target or intervention point if metabolites downstream of the target are lower enriched as the variability threshold.

Identified metabolites can be mapped onto reference pathways if the library provides KEGG IDs. Metabolites with no matching reference spectrum or identified metabolites with no KEGG identifier are mapped on the pathway based on MID similarity. As groups of similar metabolites tend to form clusters in which each metabolite is connected to all the others, the introduction of reference pathways enables the option to remove redundant connections between similar metabolites and preserve pathway topology.

MIAMI provides an interactive, dynamic force directed network visualization with an extensive set of visualization options. Additionally, MID and quantification data, as well as meta-data (deposited in the reference library) can be displayed for every detected metabolite.

MIAMI provides some additional features to facilitate data analysis, like netCDF import, ion chromatographic deconvolution, calculation of retention index based on mix of alkanes or similar set of reference compounds and import of reference libraries in MSL format. Additionally, all date can be exported in various formats.

## 3 Implementation

We implemented the data analysis back-end in C++17 using MetaboliteDetector and NTFD libraries for GCMS data processing. The front-end is implemented in Qt5 and data visualization in JavaScript using D3.js library (Bostock *et al.*, 2011). MIAMI is available at as Linux binary with graphical user interface or command line tool. Network visualization is also available as web application with HTML5 user interface.

## 4 Conclusion

Bridging the gap between non-targeted and targeted data processing of stable-isotope labeled MS data is essential for the interpretation of metabolic changes in biological processes. With MIAMI, we combine targeted and non-targeted approaches to generate metabolic networks and propose potential metabolic targets based on MID variability. We extended MID similarity-based network visualization with reference pathways. This enables the inclusion of non-targeted detected metabolites into a known biological context. MIAMI also provides a highly customizable and dynamic network visualization to aid data interpretation. The extensive export options enable further data processing with other data processing software.

*Financial Support*: none declared.

*Conflict of Interest*: none declared.
